# Caregivers' perspectives on lecanemab use for Alzheimer's disease: A national survey in China

**DOI:** 10.1002/alz.70680

**Published:** 2025-09-21

**Authors:** Shuai Liu, Shiyu Fan, Jinghuan Gan, Wang Liao, Qin Chen, Xia Li, Jiewen Zhang, Xiaochun Chen, Yong Ji

**Affiliations:** ^1^ Department of Neurology Huanhu Hospital Affiliated to Tianjin Medical University, Tianjin Key Laboratory of Cerebrovascular and Neurodegenerative Diseases Tianjin China; ^2^ Department of Neurology Beijing Friendship Hospital, Capital Medical University Xicheng District Beijing China; ^3^ Department of Neurology Institute of Neuroscience Key Laboratory of Neurogenetics and Channelopathies of Guangdong Province and the Ministry of Education of China, The Second Affiliated Hospital, Guangzhou Medical University Guangzhou China; ^4^ Department of Neurology West China Hospital of Sichuan University Chengdu Sichuan China; ^5^ Department of Psychogeriatrics Shanghai Mental Health Center Shanghai Jiaotong University School of Medicine Shanghai China; ^6^ Department of Neurology Zhengzhou University People's Hospital (Henan Provincial People's Hospital), Zhengzhou China; ^7^ Department of Neurology Fujian Medical University Union Hospital, Fujian Key Laboratory of Molecular Neurology and Institute of Neuroscience, Fujian Medical University Fuzhou Fujian China

**Keywords:** Alzheimer's disease, attitude, informal caregivers, lecanemab

## Abstract

**INTRODUCTION:**

Caregivers' decisions significantly influence Alzheimer's disease progression, yet research on the benefits of disease‐modifying therapy (DMT) from their perspective is limited.

**METHODS:**

This cross‐sectional survey included 345 informal caregivers of lecanemab‐treated patients. We collected online questionnaires from 37 tertiary hospitals across 31 provinces/autonomous regions/municipalities in China (2024/06/24 ∼ 2024/12/24).

**RESULTS:**

Approximately 94.5% of the caregivers opined that the burden of care did not intensify (including remaining constant or being alleviated) subsequent to the administration of lecanemab, among which 25.8% of the caregivers stated that the burden of care was mitigated. Those caregivers with a higher annual family income (≥¥400,000, *p *< 0.01) and filial caregivers exhibited greater confidence in the therapeutic efficacy of lecanemab.

**DISCUSSION:**

Most caregivers hold a positive attitude toward lecanemab, particularly filial caregivers. The application of lecanemab may alleviate the care burden on caregivers.

**Highlights:**

- We conducted the first China national survey on Alzheimer's disease (AD) caregiver decision‐making and lecanemab experience, collecting 345 questionnaires.
- The majority of caregivers in China hold a favorable attitude toward lecanemab.
- Higher education, younger age, and filial relationships are associated with reduced caregiver burden.
- Higher income is linked to increased caregiver confidence and better lecanemab adherence.
- There is a low adoption rate of blood‐based AD biomarkers in caregiver decisions.

## 1 BACKGROUND

Alzheimer's disease (AD) is a neurodegenerative disorder characterized by the presence of extracellular plaques composed of amyloid beta (Aβ) and intracellular neurofibrillary tangles (NFTs) containing hyperphosphorylated tau (p‐tau) protein.^1^, ^2^ The epidemiology of AD is closely related to that of all‐cause dementia, with AD being one of the most common causes of dementia.^3^ The overall crude prevalence rate of dementia among individuals ≥65 years of age in China is 9.1%.^4^ It is projected that the global number of people with all‐cause dementia will increase from 50 million in 2010 to 113 million in 2050.^5^ Currently, several disease‐modifying therapies (DMTs) have been approved, which can effectively enhance the quality of life for patients with early‐stage AD and significantly slow disease progression.^6^, ^7^, ^8^, ^9^ In a Phase III trial of patients with early AD with Aβ and tau pathology, donanemab significantly attenuated clinical progression over 76 weeks. In the low/moderate tau burden population, the mean change from baseline in Integrated Alzheimer's Disease Rating Scale (iADRS) scores at week 76 was −6.02 (95% confidence interval [CI]: −7.01 to −5.03) in the donanemab group compared with −9.27 (95% CI: −10.23 to −8.31) in the placebo group, yielding a between‐group difference of 3.25 (95% CI: 1.88–4.62; *p* < 0.001), indicating a 35.1% reduction in disease progression rate (95% CI: 19.90%–50.23%).^6^ The 221AD302 Phase 3 Study of Aducanumab in Early AD (EMERGE) study found that the difference in Clinical Dementia Rating scale Sum‐of‐Boxes (CDR‐SB) scores between the high‐dose aducanumab group and the placebo group was −0.39 (95% CI, −0.69 to −0.09; *p* = 0.012; a 22% reduction).^7^ The BAN2401‐G000‐201 study found that there was a lecanemab‐placebo difference in favor of treatment by 27% and 30% on Alzheimer's Disease Composite Score (ADCOMS), 56% and 47% on Alzheimer's Disease Assessment Scale‐Cognitive Subscale (ADAS‐Cog14), and 33% and 26% on CDR‐SB versus placebo according to Bayesian and frequentist analyses, respectively.^8^ In patients with early AD, lecanemab reduced amyloid biomarker levels. At 18 months, the adjusted least squares mean showed that the change in CDR‐SB score from baseline in the lecanemab group was 1.21, compared with 1.66 in the placebo group (between‐group difference: −0.45; 95% CI: −0.67 to −0.23; *p* < 0.001).^9^ Lecanemab is an anti‐Aβ monoclonal antibody approved by U.S. Food and Drug Administration (FDA) for the treatment of early AD (including mild cognitive impairment [MCI] and mild dementia stages). Lecanemab specifically binds to soluble Aβ protofibrils with high affinity, leading to a significant reduction in Aβ deposition in the brain and effectively slowing the decline of cognitive function.^9^, ^10^ The Phase III clinical trial of lecanemab demonstrated a significant 38% reduction in caregiver burden as assessed by the Zarit Burden Interview (ZBI) over an 18‐month period.^11^

Patients with AD gradually lose the ability to form new memories and handle daily tasks.^12^, ^13^ Consequently, family members often take on the role of caregivers to support the patient's mental well‐being and overall quality of life.^14^ In addition, current therapeutic interventions for AD through slow pathological progression (e.g., reduction of Aβ deposition) rather than reversing existing symptoms.^15^ Currently, there is no cure for AD.^16^ Therapeutic interventions focus primarily on symptom management and deceleration of disease progression.^17^ Consequently, the quality of AD management is further influenced by caregivers' decision‐making patterns (e.g., decisions about care and treatment) and psychological attitudes (e.g., perceived caregiving burden), which may influence patient outcomes.^18^, ^19^

Lecanemab was first approved for marketing by the National Medical Products Administration of China in January 2024, and officially entered the Chinese market in June of the same year. Given that lecanemab is the first DMT for AD available in China, there is currently a dearth of studies systematically evaluating the benefits it brings to Chinese AD patients and their families from the caregivers' perspective. Hence, it is urgently imperative to carry out a caregiver‐centered, multi‐center, large‐sample cross‐sectional investigation to comprehensively analyze caregivers' acceptance willingness toward lecanemab and its current application status, thereby providing evidence‐based grounds for enhancing comprehensive dementia management strategies and facilitating the scientific formulation of national‐level prevention and control policies for AD.

## 2 METHODS

### 2.1 Study design

We conducted a multi‐center, cross‐sectional survey across China, collecting data through an online questionnaire. This study was initiated by the Cognitive Disorders Group of the Chinese Society of Neurology, Chinese Medical Association, and was carried out in 37 tertiary hospitals across 31 provinces, municipalities, and autonomous regions. The recruitment for this study began on June 24, 2024, and ended on December 24, 2024, enrolling a total of 345 informal caregivers of patients who had received lecanemab treatment. Inclusion criteria: (1) Caregivers 18 years of age or older. (2) Living with the patient and closely involved in the patient's care for at least 1 year. This includes meeting the patient's daily needs, supervising medication, accompanying the patient to medical appointments, and maintaining communication with hospital staff during the patient's hospitalization. (3) Caregivers are required to spend at least 1 h each day in face‐to‐face communication with the patient. Exclusion criteria: (1) Anyone diagnosed with a mental disorder (except nicotine dependence). (2) Those unable to communicate normally. All participants were recruited after providing written informed consent. This study was conducted in accordance with the Declaration of Helsinki and its amendments and was approved by the ethics committee (JH‐ERB‐2021‐012).

**RESEARCH IN CONTEXT**

1. **Systematic review**: As China's first approved Alzheimer's disease (AD)–modifying therapy, lecanemab may alleviate AD family burdens, yet caregiver‐focused evidence remains scarce. Therefore, it is urgently necessary to conduct a large‐scale, multicenter, cross‐sectional survey centered on caregivers to deeply analyze their willingness to accept lecanemab and its actual application status.
2. **Interpretation**: This cross‐sectional survey of 345 Chinese caregivers of lecanemab‐treated early AD patients revealed majority endorsement of therapeutic benefits. Higher education, younger age, and filial relationships predicted reduced burden. Higher income boosts caregiver confidence and lecanemab adherence. Despite the high accuracy of blood‐based AD biomarkers, their low adoption (20%) underscores the need for increased caregiver education to enhance awareness and facilitate early AD detection and intervention
3. **Future directions**: These findings inform evidence‐based policymaking to alleviate caregivers' socioeconomic burdens. Future longitudinal studies should examine financial strain and psychological distress determinants among Chinese familial caregivers.

### 2.2 Study measurements

Based on previous literature (recommendations for the DMTs of early AD) and the advice of cognitive impairment experts (Jianping Jia et al.),^20^ we designed a questionnaire for caregivers to fill out. The questionnaire consists of four parts: basic information of the caregivers, their understanding and decision‐making regarding AD diagnosis, their understanding and decision‐making about lecanemab, and their feelings about using lecanemab. It involves 36 items and is expected to take 10 min to complete. The specific contents are as follows:

1. Caregiver demographics profiling. This study collects caregivers' demographic and socioeconomic characteristics profiles encompassing name, age, caregiving duration, geographic region, gender, marital status, educational attainment, occupational status, income sources, annual household income, and kinship relationship to the patient.
2. Health literacy regarding AD. It investigates caregivers' disease literacy regarding AD, including their information acquisition pathways, comprehension of diagnostic methods, and their acceptance of various diagnostic methods.
3. Understanding and decision‐making regarding lecanemab. It assesses caregivers' pharmacological literacy concerning lecanemab's therapeutic mechanisms, administration protocols, treatment expenditures, clinical effectiveness, and safety profiles, alongside decision‐making determinants for therapy adoption and expectations regarding next‐generation DMTs.
4. Caregiver experiences with lecanemab therapy. It documents the duration of lecanemab treatment, accessibility of obtaining lecanemab, whether expected effects were achieved, the presence of adverse reactions, changes in caregiving burden, willingness to continue lecanemab therapy, and underlying reasons, as well as patient cooperation levels and caregiver trust in healthcare providers.

### 2.3 Statistics

Descriptive statistics were used to report the characteristics of the participants, with categorical variables presented as frequencies and percentages, and continuous variables as means and SDs. In the univariate analysis, the Cochran–Armitage trend test was used to compare the differences between groups for binary variables, the Pearson chi‐square test was used for unordered categorical variables (with Fisher's exact test used when the expected count was <1), and the Kendall rank correlation analysis was used for ordered categorical variables. Multivariate analysis using ordered logistic regression was conducted to identify the influencing factors of caregivers' decision‐making, with independent variables including the caregivers' gender, age, education level, marital status, region, annual household income, and relationship with the patient. In all statistical tests, *p* < 0.05 was considered statistically significant.

## 3 RESULTS

### 3.1 Participant characteristics

A total of 345 family caregivers of lecanemab users who met the inclusion criteria were recruited for this study. Participants had a mean age of 50.85 years, with 93.6% being married (Table 1). The mean annual household income was about ¥390,000 ($53,412). The expenditure on the medication for the patients' families was ≈¥13,733 ($1881). Filial caregivers (patients' adult offspring) constituted the predominant demographic cohort in this study, representing most participants. The mean caregiving duration across the sample was 2.36 years.

**TABLE 1:** Characteristics of participants (*n* = 345).

Characteristic	Proportion (%)
Gender	
Male	151 (43.8)
Female	194 (56.3)
Age, years	50.85 ± 12.96
Mean ± SD	50.85 ± 12.96
Median (range)	50 (23∼88)
Marital status	
Unmarried	10 (2.9)
Married	323 (93.6)
Divorced	12 (3.5)
Widowed	0 (0)
Education level, years	14.1
Illiterate	2 (0.5)
Primary education	17 (4.9)
Lower secondary education	37(10.7)
Upper secondary education	96 (27.8)
Tertiary education	193 (55.9)
Region	
South	65 (18.8)
North	280 (81.2)
Occupation	
Self‐employed individual	47 (13.6)
Office worker	46 (13.3)
Managerial cadre	43 (12.4)
Worker	37 (10.7)
The relationship with patients	
Adult offspring	173 (50.1)
Spouse	111 (32.2)
Parents	22 (6.4)
Friend	27 (7.8)
Sibling	8 (2.3)
Others	4 (1.2)
Annual household income (¥10,000)	
Mean ± SD	39.05 (27.82)
Median (range)	40 (10 ∼ 300)
Family income sources	
Salary	233 (67.5)
Pension	177 (51.3)
Investment	106 (30.7)
Family support	120 (34.8)
Actual expenditure of lecanemab (yuan/month)	13733 ± 5015
Actual usage duration of lecanemab (months)	3.49 ± 2.99
Caregiving time (years)	2.36 ± 1.46

Abbreviation: SD, standard deviation.

### 3.2 Caregiver's knowledge/understanding about AD or lecanemab

Approximately 51.6% of caregivers reported a basic understanding of AD, whereas only 5.8% demonstrated comprehensive familiarity with AD diagnostic criteria. An additional 41.7% exhibited partial awareness of diagnostic procedures (Table S1). Offline medical institutions served as the primary information source for AD‐related knowledge among caregivers (Figure S1). Regarding diagnostic preferences, positron emission tomography (PET) was selected by 91.3% of participants, contrasting with substantially lower utilization rates for blood tests (20%) and CSF analyses (17.7%) (Table S2). When therapeutic options were evaluated, drug efficacy emerged as the predominant concern for 66.4% of caregivers, with safety considerations prioritized by 33.6% of respondents (Table S2).

Only 8.7% of caregivers demonstrated an in‐depth understanding of lecanemab's mechanism of action, whereas 53.3% had a relatively good understanding (Table S3). Approximately 82.9% were well informed about the administration method and interval of lecanemab, 84.6% about its price, 72.7% about expected outcomes, and 74.8% about adverse reactions (Table S3).

### 3.3 The driving factors for caregivers to choose lecanemab

Caregivers demonstrated substantial trust in prescribing physicians, with 42.9% expressing complete trust and 52.46% expressing considerable trust (Figure S2). The majority of caregivers opted for lecanemab therapy, driven predominantly by clinician recommendations (82.9%) (Table S2).

### 3.4 Caregiver experiences with lecanemab therapy

**FIGURE 1:**
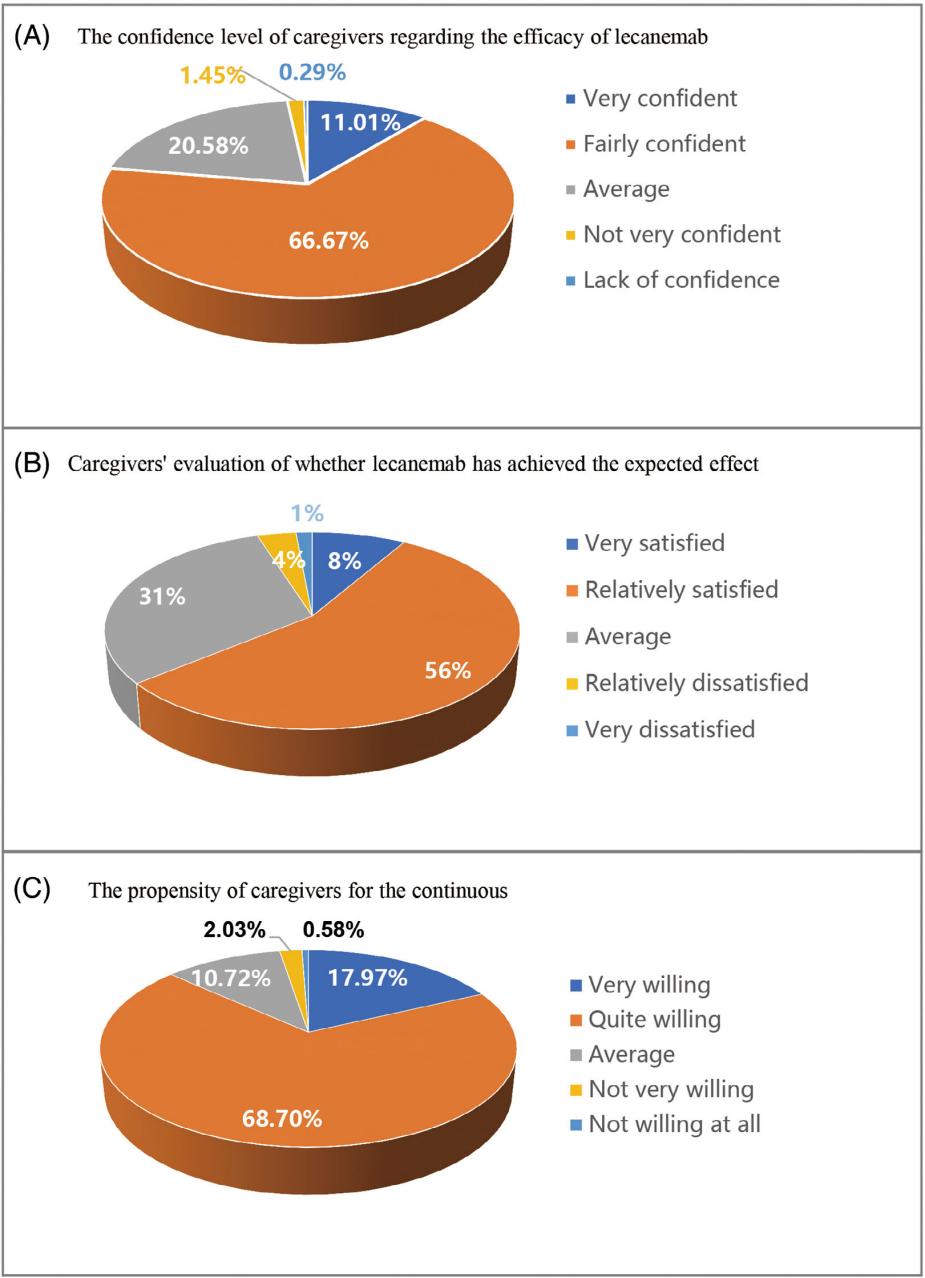
Caregiver experiences with lecanemab therapy.

Approximately 77.68% of caregivers expressed high or moderate confidence in the efficacy of lecanemab (Figure 1A). Following a period of treatment, 64% of caregivers reported that lecanemab had achieved the expected therapeutic effect (Figure 1B), and 86.7% were willing to continue using lecanemab (Figure 1C). The reasons for these caregivers' willingness to continue administering lecanemab include adhering to the guidance of clinicians regarding the completion of the treatment regimen (64.9%) and the favorable safety profile of lecanemab (49.4%), among others (Table S2).

Nearly 87% of caregivers believed patients were highly or moderately cooperative with the treatment regimen (Figure S3). Regarding lecanemab treatment accessibility, over 50% of caregivers reported very or relatively convenient access to the medication. (Figure S4).

### 3.5 Treatment‐emergent adverse events during lecanemab administration

About 19.4% of the patients experienced adverse reactions during the medication process, with the most common being fever (11.9%), followed by dizziness (5.5%) and headache (3.8%) (Table S4).

### 3.6 Changes in caregiving burden following lecanemab initiation

Within less than 6 months after the initiation of lecanemab treatment, ≈68.7% of caregivers opined that no significant change occurred in the care burden; 25.8% of caregivers reported an alleviation of the care burden, whereas ≈5.5% of caregivers stated that their care responsibilities had augmented (Table S4).

### 3.7 Caregivers' expectations and suggestions for new DMTs

According to the study results, the majority of patients (85.8%) are now taking lecanemab in hospital wards, which is also where caregivers expect it to be administered (75.7%) (Table S5). Day infusion centers (14.8%) and community clinics (13.9%) are also being evaluated as future DMT administration sites. The majority of participants hope that oral (84.9%) and subcutaneous injection (49.9%) dosage forms will become available in the future. Caregivers anticipate that the medicine administration interval will be 1 month (71.9%), with a total duration of 1 year (62.3%) (Table S5). Furthermore, ≈89.8% of caregivers are concerned about the inclusion of lecanemab in social health insurance coverage (Table S5).

### 3.8 Univariate analysis of the association between demographic characteristics and caregivers' decisions

In the assessment of confidence in lecanemab efficacy, those with a higher annual family income (*p *= 0.001) and those with a parent–child relationship with the patient (*p *= 0.001) showed significantly enhanced confidence (Table S6). Higher satisfaction with lecanemab efficacy was reported among participants from households with higher annual household incomes (*p* < 0.001) and notably by filial caregivers (*p *= 0.040), both demonstrating significantly elevated satisfaction scores (Table S7). Married participants (*p* = 0.006), caregivers from higher‐income households (*p* = 0.014), and filial caregivers (*p* = 0.011) demonstrated significantly greater willingness to continue lecanemab therapy (Table S8). In terms of the understanding of AD, individuals with bachelor's degrees or above (*p *< 0.001) and filial caregivers (*p *< 0.001) have a deeper understanding of the disease (Table S9).

### 3.9 Multivariate ordered logistic regression analysis of factors influencing caregivers' decision‐making

Regarding AD comprehension, filial caregivers demonstrated significantly greater disease comprehension relative to spousal caregivers (odds ratio [OR] = 3.23, 95% CI: 1.85–5.88; *p* < 0.001) and other caregivers (OR = 2.63, 95% CI: 1.43–4.76; *p* = 0.002). Elderly caregivers (OR = 2.02, 95% CI: 1.04–3.94; *p* = 0.039) and those with tertiary education (OR = 2.04, 95% CI: 1.31–3.18; *p* = 0.002) also showed enhanced disease understanding (Table 2).

**TABLE 2:** Multivariate ordinal logistic regression analysis of factors influencing caregivers' cognitive level of Alzheimer's disease.

					OR		
Factors	*β*	SE	Wald's chi‐square value	*p*	Point estimation	Lower confidence limit	Upper confidence limit
Gender							
Male vs Female	−0.03	0.21	0.02	0.901	0.97	0.64	1.48
Age							
Middle‐aged v. youth	0.53	0.28	3.57	0.059	1.7	0.98	2.93
The elder vs youth	0.7	0.34	4.25	0.039^*^	2.02	1.04	3.94
Education level							
Tertiary education vs non‐tertiary education	0.71	0.23	9.82	0.002^*^	2.04	1.31	3.18
Marital status							
Married vs unmarried	−0.4	0.65	0.37	0.544	0.67	0.19	2.41
Other vs unmarried	−0.69	0.83	0.69	0.406	0.5	0.1	2.57
Annual household income (¥10,000)							
20–39 vs <20	−0.2	0.3	0.44	0.506	0.82	0.46	1.47
40–59 vs <20	0.26	0.31	0.7	0.402	1.29	0.71	2.35
>59 vs <20	−0.02	0.34	0	0.947	0.98	0.5	1.92
Relationship with the patient							
Spouse vs adult offspring	−1.19	0.29	16.49	< 0.001^**^	3.23	1.85	5.88
Other vs adult offspring	−0.96	0.31	9.92	0.002^*^	2.63	1.43	4.76

Abbreviations: OR, odds ratio; SE, standard error.

* *p *< 0.05.

** *p* < 0.001.

Lecanemab efficacy confidence assessments identified three significant predictors: elderly caregivers exhibited stronger confidence (OR = 3.06, 95% CI: 1.47–6.38; *p* = 0.003), high annual household income (¥400,000–¥590,000 group: OR = 2.48, 95% CI: 1.29–4.77, *p* = 0.007; >¥590,000 group: OR = 2.59, 95% CI: 1.24–5.45, *p* = 0.012) relative to the <¥200,000 group, and filial versus spousal caregivers (OR = 2.70, 95% CI: 1.47–5.00; *p* = 0.001) (Table 3).

**TABLE 3:** Multivariate ordered logistic regression analysis of factors influencing the caregivers' confidence in the efficacy of lecanemab.

					OR		
Factors	*β*	SE	Wald's chi‐square value	*p*	Point estimation	Lower confidence limit	Upper confidence limit
Gender							
Male vs female	−0.18	0.23	0.62	0.431	0.83	0.53	1.31
Age							
Middle‐aged vs youth	0.27	0.30	0.81	0.367	1.32	0.73	2.38
The elder vs. youth	1.12	0.38	8.86	0.003^*^	3.06	1.47	6.38
Education level							
Tertiary education vs non‐tertiary education	0.31	0.25	1.54	0.214	1.36	0.84	2.20
Marital status							
Married vs unmarried	0.07	0.70	0.01	0.920	1.07	0.27	4.20
Other vs unmarried	−0.40	0.89	0.21	0.650	0.67	0.12	3.82
Annual household income (¥10,000)							
20–39 vs <20	0.41	0.32	1.66	0.198	1.51	0.81	2.82
40–59 vs <20	0.91	0.33	7.37	0.007^*^	2.48	1.29	4.77
>59 vs <20	0.95	0.38	6.34	0.012^*^	2.59	1.24	5.45
Relationship with the patient							
Spouse vs adult offspring	1.01	0.32	10.23	0.001^*^	2.70	1.47	5.00
Other vs adult offspring	0.14	0.33	0.17	0.679	1.15	0.60	2.17

Abbreviations: OR, odds ratio; SE, standard error.

* *p* < 0.05.

Those with an annual household income of ¥400,000–¥590,000 (OR = 3.58, 95% CI: 1.92–6.67; *p* < 0.001) and >¥590,000 (OR = 3.94, 95% CI: 1.94–8.03; *p* < 0.001) compared to those with an income of less than ¥200,000, and filial caregivers (OR = 2.44, 95% CI: 1.37–4.35; *p *= 0.002) compared to spouses, have a higher satisfaction with the efficacy of lecanemab (Table 4).

**TABLE 4:** Multivariate ordered logistic regression analysis of factors influencing caregivers' satisfaction with the efficacy of lecanemab.

					OR		
Factors	*β*	SE	Wald's chi‐square value	*p*	Point estimation	Lower confidence limit	Upper confidence limit
Gender							
Male vs female	0.25	0.22	1.30	0.254	1.28	0.84	1.97
Age							
Middle‐aged vs youth	0.11	0.29	0.15	0.698	1.12	0.64	1.96
The elder vs youth	0.57	0.35	2.61	0.106	1.77	0.89	3.53
Education level							
Tertiary education vs non‐tertiary education	−0.24	0.23	1.02	0.312	0.79	0.50	1.25
Marital status							
Married vs unmarried	0.39	0.66	0.35	0.554	1.48	0.41	5.37
Other vs unmarried	−0.31	0.84	0.14	0.714	0.73	0.14	3.83
Annual household income (¥10,000)							
20–39 vs <20	0.40	0.30	1.80	0.180	1.50	0.83	2.70
40–59 vs <20	1.27	0.32	16.04	< 0.001^**^	3.58	1.92	6.67
>59 vs <20	1.37	0.36	14.27	< 0.001^**^	3.94	1.94	8.03
Relationship with the patient							
Spouse vs adult offspring	0.90	0.30	9.18	0.002^*^	2.44	1.37	4.35
Other vs adult offspring	0.15	0.31	0.23	0.635	1.16	0.63	2.13

Abbreviations: OR, odds ratio; SE, standard error.

* *p* < 0.05.

** *p* < 0.001.

In evaluating caregivers' willingness to continue lecanemab treatment for patients, the results indicate that elderly caregivers (OR = 2.20, 95% CI: 1.05–4.64; *p *= 0.038) and those with an annual household income between ¥400,000 and ¥590,000, compared to those with an income <¥200,000 (OR = 2.57, 95% CI: 1.31–5.05; *p *= 0.006), exhibit greater propensity to endorse therapeutic continuation (Table 5).

**TABLE 5:** Multivariate ordered logistic regression analysis of factors influencing the willingness of caregivers to continue using lecanemab.

					OR		
Factors	*β*	SE	Wald's chi‐square value	*p*	Point estimation	Lower confidence limit	Upper confidence limit
Gender							
Male vs female	−0.06	0.24	0.07	0.786	0.94	0.59	1.49
Age							
Middle‐aged vs youth	0.59	0.31	3.61	0.057	1.80	0.98	3.31
The elder vs youth	0.79	0.38	4.31	0.038^*^	2.20	1.05	4.64
Education level							
Tertiary education vs non‐tertiary education	−0.30	0.25	1.39	0.239	0.74	0.46	1.22
Marital status							
Married vs unmarried	0.85	0.68	1.55	0.213	2.33	0.62	8.84
Other vs unmarried	0.44	0.89	0.24	0.623	1.55	0.27	8.86
Annual household income (¥10,000)							
20–39 vs <20	−0.06	0.34	0.03	0.870	0.95	0.49	1.82
40–59 vs <20	0.94	0.34	7.53	0.006^*^	2.57	1.31	5.05
>59 vs <20	0.46	0.39	1.43	0.232	1.59	0.74	3.39
Relationship with the patient							
Spouse vs adult offspring	0.38	0.32	1.40	0.237	1.45	0.78	2.70
Other vs adult offspring	0.09	0.34	0.08	0.784	1.10	0.57	2.13

Abbreviations: OR, odds ratio; SE, standard error.

* *p* < 0.05.

### 3.10 Multivariate ordered logistic regression analysis of influencing factors of caregiver burden changes

Using changes in caregiver burden as the dependent variable and various potential influencing factors as independent variables, we conducted an ordered logistic regression analysis to identify factors influencing changes in caregiver burden. Tertiary‐educated caregivers (vs non‐tertiary education: OR = 1.66, 95% CI: 1.00–2.76; *p* = 0.048), those 20–30 years of age (vs <20 years: OR = 2.02, 95% CI: 1.03–3.98; *p* = 0.041), and filial caregivers (vs others: OR = 2.33, 95% CI: 1.99‐–4.55; *p* = 0.014) exhibited significantly elevated odds of burden alleviation (Table 6).

**TABLE 6:** Multivariate ordered logistic regression analysis of influencing factors of caregiver burden changes.

					OR		
Factors	*β*	SE	Wald's chi‐square value	*p*	Point estimation	Lower confidence limit	Upper confidence limit
Gender							
Male vs female	0.01	0.24	0.00	0.956	1.01	0.63	1.63
Age							
Middle‐aged vs youth	−0.26	0.32	0.62	0.433	0.78	0.41	1.46
The elder vs youth	−0.53	0.39	1.81	0.179	0.59	0.27	1.28
Education level							
Tertiary education vs non‐tertiary education	0.51	0.26	3.90	0.048^*^	1.66	1.00	2.76
Marital status							
Married vs unmarried	−0.22	0.75	0.09	0.768	0.80	0.19	3.46
Other vs unmarried	−0.71	0.95	0.57	0.453	0.49	0.08	3.13
Annual household income (¥10,000)							
20–39 vs <20	0.71	0.35	4.16	0.041^*^	2.02	1.03	3.98
40–59 vs <20	0.41	0.35	1.39	0.239	1.51	0.76	2.98
> 59 vs <20	−0.14	0.38	0.13	0.714	0.87	0.41	1.84
Relationship with the patient							
Spouse vs adult offspring	−0.51	0.33	2.38	0.123	0.60	0.31	1.15
Other vs adult offspring	0.84	0.34	6.07	0.014^*^	2.33	1.19	4.55

Abbreviations: OR, odds ratio; SE, standard error.

* *p* < 0.05.

## 4 DISCUSSION

In this study, the proportion opting for blood biomarker assays (20%) was lower. This suggests that caregivers' cognition and acceptance of blood‐based AD biomarkers remain conspicuously limited. Previous studies have affirmed that blood biomarkers of AD possess higher sensitivity, specificity, and accuracy.^21^, ^22^ One study revealed that plasma p‐tau217 can discriminate AD from other neurodegenerative diseases, with a higher accuracy than established MRI‐based biomarkers.^22^ The diagnostic performance of the ratio of plasma p‐tau217 to non‐phosphorylated tau is equivalent to or superior to that of clinical CSF tests, enhancing the accessibility of accurate AD diagnosis.^23^ Therefore, it is imperative to intensify patient education and publicity regarding blood biomarkers of AD and enhance caregivers' awareness and acceptance. This will facilitate the extensive application of blood biomarkers across health care settings, allowing for the identification of high‐risk AD patients among individuals with normal cognition or MCI, thus offering critical support for timely diagnosis and therapeutic interventions in AD management.

In addition to the clinical and biological outcomes of lecanemab, the perspective of caregiver burden is recognized increasingly as a critical metric for evaluating the efficacy of DMTs in AD. Previous studies have found that the burden on caregivers is related to the annual household income of patients with AD and the severity of the disease.^24^, ^25^, ^26^, ^27^, ^28^ As highlighted in recent research, the concept of meaningful benefit in AD treatment extends beyond traditional cognitive and functional measures to encompass the holistic impact on caregivers, including reductions in caregiving burden and improvements in quality of life.^29^ The Phase III CLARITY AD trial demonstrated that lecanemab significantly slowed health‐related quality of life (HRQoL) decline (38% reduction in progression burden, measured by ZBI) and alleviated caregiver burden compared to placebo.^11^ Our findings align with the HRQoL result from CLARITY AD, with 94.5% of caregivers reporting no aggravation of their care burden (remaining unchanged or mitigated) within 6 months of lecanemab treatment, whereas 25.8% reported a decrease in their burden. This suggests that lecanemab may mitigate the progressive demands placed on caregivers, even in the early stages of treatment. These findings indicate that within a relatively short treatment period, lecanemab demonstrates clinical intervention value for the caregiving system in AD management in China.

Our multivariate regression analysis identified three significant predictors of caregiver burden alleviation. First, caregivers with tertiary education demonstrated significantly greater burden reduction (OR = 1.66, 95% CI: 1.00–2.76; *p* = 0.048) compared to those without higher education, suggesting a 66% increased probability of improved outcomes. Young adult caregivers 20–30 years of age showed a 2.02‐fold greater probability of burden reduction relative to caregivers younger than 20 (*p *= 0.041). Notably, filial caregivers manifested superior burden mitigation outcomes with an OR of 2.33 (*p *= 0.014) compared to other caregivers. These results likely reflect the unique challenges faced by filial caregivers in reconciling professional obligation with familial responsibilities, chronic psychological distress from witnessing parental cognitive decline.^30^ These compounding stressors may explain why filial caregivers experience more significant burden reduction when utilizing lecanemab treatment regimens, as the therapy potentially alleviates multiple aspects of their caregiving strain.

The univariate analysis demonstrated that caregivers with elevated annual household incomes exhibited enhanced therapeutic confidence, greater satisfaction with lecanemab efficacy, and stronger propensity for treatment continuation. Multivariate analysis using ordered logistic regression identified two significant predictors of treatment satisfaction: (1) caregivers with annual household incomes between ¥400,000 and ¥590,000, and those exceeding ¥590,000; and (2) filial caregivers. Furthermore, advanced age and mid‐tier income status (¥400,000–¥590,000) emerged as positive predictors for treatment continuation. These results substantiate a robust correlation between caregiver socioeconomic status and treatment satisfaction. This study revealed that the mean annual household income of participants was ¥390,000, with monthly lecanemab expenditures averaging ¥13,733. Comparative data from international cohorts demonstrate cost variations: patients with early‐stage AD incurred €10,558 (France)^31^ and $9431 (USA)^32^ in annual direct medical costs. Another cross‐sectional study involving 1675 Chinese AD patients showed that 49% of the patients had annual medical expenses below ¥10,000, and 34.93% had annual medical expenses ranging from ¥10,000 to ¥24,000.^24^ This disparity may be attributed to the elevated pricing of DMTs in China. However, beyond direct medical costs, substantial indirect costs from caregiver burden and productivity loss, alongside intangible costs of reduced quality of life among caregivers and families, warrant critical evaluation.^33^, ^34^, ^35^ These indirect expenditures potentially account for a considerable component of comprehensive socioeconomic burdens linked to AD and may also reflect the most highly valued outcomes for caregivers and patients.^36^ A health economic modeling study based on the Phase III CLARITY AD trial demonstrated that lecanemab combined with standard of care (SOC) reduced total health care expenditures by ¥1,152,772 from the payer perspective, while achieving ¥1,989,509 in societal cost savings through comprehensive economic evaluation in Japan.^37^ Another study on the cost‐effectiveness of AD treatments found that lecanemab extends patient survival by over 0.6 years, with a societal value per quality‐adjusted life year (QALY) reaching ¥1.94 million to ¥4.68 million (Japan) or $19,000 to $37,000 (USA).^38^ In view of this, it is imperative to establish an integrated health care security framework encompassing direct medical expenditure governance and implicit socio‐economic burden distribution, which can systematically mitigate the financial strain on AD caregivers and societal economic burdens through dual‐cost regulatory mechanisms.

This study revealed optimization demands from caregivers across multiple parameters, including administration setting, dosage form, treatment frequency, duration, and cost considerations. Our findings suggest that 83.7% of caregivers expected to receive lecanemab treatment in inpatient wards, primarily due to safety monitoring requirements. With the increasing refinement and adoption of lecanemab treatment protocols, day‐care infusion centers and community clinics are emerging as pivotal administration venues. This transition substantially enhances patient and caregiver convenience by alleviating burdens associated with hospitalization/discharge procedures, appointment scheduling, and transportation logistics. The established intravenous administration route for lecanemab reflects careful consideration of pharmacokinetic properties, target specificity, and clinical trial evidence.^39^ Nevertheless, our findings reveal significant caregiver preference for oral (72.3%) and subcutaneous (65.1%) formulations, which clinical studies suggest may reduce health care costs and simplify treatment administration.^40^, ^41^ The GRADUATION trial evaluated the pharmacodynamic effects of weekly subcutaneous administration of 255 mg gantenerumab in patients with early symptomatic AD.^42^ This pioneering study authorized standardized‐trained, investigator‐certified non‐medical caregivers to administer therapeutics domestically, effectively alleviating health care system strain while enhancing treatment accessibility and reducing clinical visit frequency.^42^, ^43^ Regarding treatment frequency and duration, 71.9% of caregivers preferred monthly administration over the current biweekly regimen, with most anticipating a 12‐month treatment course. These preferences underscore the need for enhanced physician–patient communication regarding the temporal dynamics of amyloid reduction with anti‐Aβ therapies. Clinical evidence demonstrates that lecanemab exhibits a characteristic treatment trajectory: amyloid reduction becomes detectable by 3 months, clinical efficacy emerges by 6 months, and amyloid clearance (defined as amyloid negativity) occurs in >80% of patients within 12–18 months.^44^ However, the recent FDA approval of monthly maintenance dosing represents an important step toward optimizing treatment intervals. Further real‐world evidence is required to establish optimal dosing frequencies that maintain treatment efficacy while improving patient and caregiver experience, to fully evaluate the impact of modified dosing regimens. Finally, cost concerns emerged prominently, with 89.8% of caregivers emphasizing the need for national health insurance coverage given the relatively high costs of the treatment. We recommend a comprehensive optimization strategy incorporating formulation development, extended dosing intervals, and reimbursement policies to establish a sustainable, patient‐centered treatment paradigm that effectively balances therapeutic efficacy with caregiver support.

This study represents the first cross‐sectional investigation to systematically evaluate caregiver perspectives on AD management, including treatment decision‐making processes and emotional experiences surrounding lecanemab use. As China undergoes rapid economic development and accelerated population aging, dementia has emerged as a critical public health challenge. This challenge aligns directly with priorities outlined in the WHO's Global Action Plan on the Public Health Response to Dementia, which positions family caregivers as the cornerstone of effective dementia care systems, urging member states to implement structural support programs and professional training for caregivers. Our findings provide empirical evidence for health policy development, particularly for DMTs in patients with early AD to mitigate caregiver burden. We recommend enhanced government investment in early dementia detection and implementation of multidimensional interventions during initial disease stages to potentially reduce both caregiver strain and dementia incidence.

There are indeed some limitations and areas for improvement in future research. Due to practical operational constraints, we were unable to conduct multi‐stage sampling to ensure a balanced geographical distribution of the sample. This study was led by voluntarily cooperating local health centers (which are usually located in urban areas), resulting in a higher number of patients from the north in the sample. It is worthy of further exploration in a larger and more balanced patient cohort. In addition, this study concentrated only on the caregivers of patients within the clinical management pathway. Consequently, the perspectives of non‐medicated patient groups were not incorporated, thereby precluding a direct comparative analysis of treatment cognition across different treatment‐status subgroups. The cross‐sectional study design has inherent limitations in evaluating the long‐term safety of drugs. Therefore, conducting longitudinal studies with multiple follow‐up time points in the future will help to more comprehensively assess the long‐term efficacy and safety profile of lecanemab, thereby providing a more reliable basis for clinical decision‐making.

In summary, our research revealed that most caregivers have a favorable view of lecanemab. Factors such as higher education level, being a younger adult caregiver, and having a filial relationship with the patient were identified as predictors that could help reduce the caregiving burden. In addition, caregivers from households with higher incomes exhibited greater confidence, satisfaction, and intention to persist with lecanemab treatment. These findings provide important information for health care policymakers to develop well‐informed strategies aimed at easing the burdens associated with caregiving.

## CONFLICT OF INTEREST STATEMENT

The authors declare no conflict of interest. Any author disclosures are available in the Supporting Information.

## CONSENT STATEMENT

All participants provided written informed consent prior to their involvement in the study. This study was approved by the Ethics Committee of Tianjin Huanhu Hospital (JH‐ERB‐2021‐012).

## Supporting information



Supporting Information

Supporting Information

## References

[1] Knopman DS , Amieva H , Petersen RC , et al. Alzheimer disease. Nat Rev Dis Primers. 2021;7:33. 33986301 10.1038/s41572-021-00269-y PMC8574196

[2] Masters CL , Bateman R , Blennow K , Rowe CC , Sperling RA , Cummings JL . Alzheimer's disease. Nat Rev Dis Primers. 2015;1:15056. 27188934 10.1038/nrdp.2015.56

[3] Boyle PA , Yu L , Wilson RS , Leurgans SE , Schneider JA , Bennett DA . Person‐specific contribution of neuropathologies to cognitive loss in old age. Ann Neurol. 2018;83:74‐83. 29244218 10.1002/ana.25123 PMC5876116

[4] Gan J , Zeng Y , Huang G , et al. The updated prevalence and risk factors of dementia in old adults in China: a cross‐sectional study. J Alzheimers dis. 2024;102:1209‐1223. 39593256 10.1177/13872877241297155

[5] Scheltens P , De Strooper B , Kivipelto M , et al. Alzheimer's disease. Lancet. 2021;397:1577‐1590. 33667416 10.1016/S0140-6736(20)32205-4 PMC8354300

[6] Sims JR , Zimmer JA , Evans CD , et al. Donanemab in early symptomatic Alzheimer disease: the TRAILBLAZER‐ALZ 2 randomized clinical trial. JAMA. 2023;330:512‐527. 37459141 10.1001/jama.2023.13239 PMC10352931

[7] Budd Haeberlein S , Aisen PS , Barkhof F , et al. Two randomized phase 3 studies of aducanumab in early Alzheimer's disease. J Prev Alzheimers Dis. 2022;9:197‐210. 35542991 10.14283/jpad.2022.30

[8] Swanson CJ , Zhang Y , Dhadda S , et al. A randomized, double‐blind, phase 2b proof‐of‐concept clinical trial in early Alzheimer's disease with lecanemab, an anti‐Aβ protofibril antibody. Alzheimers Res Ther. 2021;13:80. 33865446 10.1186/s13195-021-00813-8 PMC8053280

[9] van Dyck CH , Swanson CJ , Aisen P , et al. Lecanemab in early Alzheimer's disease. N Engl J Med. 2023;388:9‐21. 36449413 10.1056/NEJMoa2212948

[10] Wu W , Ji Y , Wang Z , et al. The FDA‐approved anti‐amyloid‐β monoclonal antibodies for the treatment of Alzheimer's disease: a systematic review and meta‐analysis of randomized controlled trials. Eur J Med Res. 2023;28:544. 38017568 10.1186/s40001-023-01512-w PMC10683264

[11] Cohen S , van Dyck CH , Gee M , et al. Lecanemab clarity AD: quality‐of‐life results from a randomized, double‐blind phase 3 trial in early Alzheimer's disease. J Prev Alzheimers dis. 2023;10:771‐777. 37874099 10.14283/jpad.2023.123

[12] Monteiro AR , Barbosa DJ , Remião F , Silva R . Alzheimer's disease: insights and new prospects in disease pathophysiology, biomarkers and disease‐modifying drugs. Biochem Pharmacol. 2023;211:115522. 36996971 10.1016/j.bcp.2023.115522

[13] 2024 Alzheimer's disease facts and figures. Alzheimers Dement. 2024;20:3708‐3821. 38689398 10.1002/alz.13809 PMC11095490

[14] Vu M , Mangal R , Stead T , Lopez‐Ortiz C , Ganti L . Impact of Alzheimer's disease on caregivers in the United States. Health Psychol Res. 2022;10:37454. 35999976 10.52965/001c.37454 PMC9392839

[15] Bazzari FH , Abdallah DM , El‐Abhar HS . Pharmacological interventions to attenuate Alzheimer's disease progression: the story so far. Curr Alzheimer Res. 2019;16:261‐277. 30827243 10.2174/1567205016666190301111120

[16] Soni U , Singh K , Jain D , Pujari R . Exploring Alzheimer's disease treatment: established therapies and novel strategies for future care. Eur J Pharmacol. 2025;998:177520. 40097131 10.1016/j.ejphar.2025.177520

[17] Passeri E , Elkhoury K , Morsink M , et al. Alzheimer's disease: treatment strategies and their limitations. Int J Mol Sci. 2022;23(22):13954. 36430432 10.3390/ijms232213954 PMC9697769

[18] Truglio‐Londrigan M , Slyer JT . Caregiver decisions along the Alzheimer's disease trajectory. Geriatr Nurs. 2019;40:257‐263. 30503603 10.1016/j.gerinurse.2018.10.015

[19] Iavarone A , Ziello AR , Pastore F , Fasanaro AM , Poderico C . Caregiver burden and coping strategies in caregivers of patients with Alzheimer's disease. Neuropsychiatr Dis Treat. 2014;10:1407‐1413. 25114532 10.2147/NDT.S58063 PMC4122550

[20] Expert Consensus Review Committee on Disease‐Modifying Treatments for Early Alzheimer′s Disease. [Recommendations for the disease‐modifying treatments of early Alzheimer's disease]. Zhonghua Nei Ke Za Zhi. 2025;64(5):385‐395. 40205735 10.3760/cma.j.cn112138-20241028-00709

[21] Yamashita K , Miura M , Watanabe S , et al. Fully automated and highly specific plasma β‐amyloid immunoassays predict β‐amyloid status defined by amyloid positron emission tomography with high accuracy. Alzheimers Res Ther. 2022;14:86. 35739591 10.1186/s13195-022-01029-0 PMC9219197

[22] Palmqvist S , Janelidze S , Quiroz YT , et al. Discriminative accuracy of plasma phospho‐tau217 for Alzheimer's disease vs. other neurodegenerative disorders. JAMA. 2020;324:772‐781. 32722745 10.1001/jama.2020.12134 PMC7388060

[23] Barthélemy NR , Salvadó G , Schindler SE , et al. Highly accurate blood test for Alzheimer's disease is similar or superior to clinical cerebrospinal fluid tests. Nat Med. 2024;30:1085‐1095. 38382645 10.1038/s41591-024-02869-z PMC11031399

[24] Li Y , Leng F , Xiong Q , et al. Factors associated with Alzheimer's disease patients' caregiving status and family caregiving burden in China. Front Aging Neurosci. 2022;14:865933. 35370609 10.3389/fnagi.2022.865933 PMC8970011

[25] Montgomery W , Goren A , Kahle‐Wrobleski K , Nakamura T , Ueda K . Alzheimer's disease severity and its association with patient and caregiver quality of life in Japan: results of a community‐based survey. BMC Geriatr. 2018;18:141. 29898679 10.1186/s12877-018-0831-2 PMC6000944

[26] Kawano Y , Terada S , Takenoshita S , et al. Patient affect and caregiver burden in dementia. Psychogeriatrics. 2020;20:189‐195. 31698515 10.1111/psyg.12487

[27] Tay LX , Ong SC , Tay LJ , Ng T , Parumasivam T . Economic burden of Alzheimer's disease: a systematic review. Value Health Reg Issues. 2024;40:1‐12. 37972428 10.1016/j.vhri.2023.09.008

[28] Lv H , Yang S , Zhang Y , et al. Caregiver burden and associated factors among informal caregivers of hospitalized elderly patients in China: a latent profile analysis. Risk Manag Healthc Policy. 2025;18:547‐559. 39990616 10.2147/RMHP.S499768 PMC11846611

[29] Elhage A , Cohen S , Cummings J , et al. Defining benefit: clinically and biologically meaningful outcomes in the next‐generation Alzheimer's disease clinical care pathway. Alzheimers Dementia. 2025;21:e14425. 10.1002/alz.14425 PMC11848336 39697158

[30] Possin KL , Dulaney S , Sideman AB , et al. Long‐term effects of collaborative dementia care on quality of life and caregiver well‐being. Alzheimers Dement. 2025;21:e14370. 39559905 10.1002/alz.14370 PMC11782176

[31] Dauphinot V , Potashman M , Levitchi‐Benea M , Su R , Rubino I , Krolak‐Salmon P . Economic and caregiver impact of Alzheimer's disease across the disease spectrum: a cohort study. Alzheimers Res Ther. 2022;14:34. 35151368 10.1186/s13195-022-00969-x PMC8841058

[32] Leibson CL , Long KH , Ransom JE , et al. Direct medical costs and source of cost differences across the spectrum of cognitive decline: a population‐based study. Alzheimers Dement. 2015;11:917‐932. 25858682 10.1016/j.jalz.2015.01.007 PMC4543557

[33] Makin C , Neumann P , Peschin S , Goldman D . Modelling the value of innovative treatments for Alzheimer's disease in the United States. J Med Econ. 2021;24:764‐769. 33989095 10.1080/13696998.2021.1927747

[34] Gustavsson A , Pemberton‐Ross P , Gomez Montero M , Hashim M , Thompson R . Challenges in demonstrating the value of disease‐modifying therapies for Alzheimer's disease. Expert Rev Pharmacoeconom Outcomes Res. 2020;20:563‐570. 10.1080/14737167.2020.1822738 32951480

[35] Wahlberg K , Winblad B , Cole A , et al. People get ready! a new generation of Alzheimer's therapies may require new ways to deliver and pay for healthcare. J Intern Med. 2024;295:281‐291. 38098165 10.1111/joim.13759

[36] El‐Hayek YH , Wiley RE , Khoury CP , et al. Tip of the iceberg: assessing the global socioeconomic costs of Alzheimer's disease and related dementias and strategic implications for stakeholders. J Alzheimers Dis. 2019;70:323‐341. 31256142 10.3233/JAD-190426 PMC6700654

[37] Igarashi A , Azuma MK , Zhang Q , et al. Predicting the societal value of lecanemab in early Alzheimer's disease in Japan: a patient‐level simulation. Neurol Ther. 2023;12:1133‐1157. 37188886 10.1007/s40120-023-00492-7 PMC10310671

[38] Tahami Monfared AA , Ye W , Sardesai A , et al. Estimated societal value of lecanemab in patients with early Alzheimer's disease using simulation modeling. neurology and therapy. 2023;12:795‐814. 36929345 10.1007/s40120-023-00460-1 PMC10195963

[39] Park J , Simpson C , Patel K . Lecanemab: a humanized monoclonal antibody for the treatment of early Alzheimer's disease. Ann Pharmacother. 2024;58:1045‐1053. 38095619 10.1177/10600280231218253

[40] Robinson RL , Rentz DM , Andrews JS , et al. Costs of early stage Alzheimer's disease in the United States: cross‐sectional analysis of a prospective cohort study (GERAS‐US)1. J Alzheimers Dis. 2020;75:437‐450. 32250304 10.3233/JAD-191212 PMC7306889

[41] Ozawa T , Franguridi G , Mattke S . Medical costs and caregiver burden of delivering disease‐modifying Alzheimer's treatments with different duration and route of administration. J Prev Alzheimers Dis. 2024;11:1384‐1389. 39350384 10.14283/jpad.2024.81 PMC11436425

[42] Boess FG , Scelsi MA , Grimmer T , et al. At‐home administration of gantenerumab by care partners to people with early Alzheimer's disease: feasibility, safety, and pharmacodynamic impact. J Prev Alzheimers Dis. 2024;11:537‐548. 38706270 10.14283/jpad.2024.60

[43] Epstein RS . Payer perspectives on intravenous versus subcutaneous administration of drugs. Clinicoecon Outcomes Res. 2021;13:801‐807. 34531668 10.2147/CEOR.S317687 PMC8439384

[44] McDade E , Cummings JL , Dhadda S , et al. Lecanemab in patients with early Alzheimer's disease: detailed results on biomarker, cognitive, and clinical effects from the randomized and open‐label extension of the phase 2 proof‐of‐concept study. Alzheimers Res & Ther. 2022;14:191. 36544184 10.1186/s13195-022-01124-2 PMC9768996

