# Supplementary material for: Caregivers' perspectives on lecanemab use for Alzheimer's disease: A national survey in China
Source: Alzheimers Dement. 2025 Sep 21;21(9):e70680. doi: 10.1002/alz.70680 (PMC12450581; doi:10.1002/alz.70680)
Supplement: Supplementary file 2 — Supporting Information [file ALZ-21-e70680-s002.docx]

**Journal**:

**Article title:** Caregivers' attitudes regarding the application of Lecanemab for Alzheimer's disease: a cross-sectional survey in China

**Author names**:

**Corresponding author affiliation and e-mail address**:

# Supplementary Material

**Supplementary Table 1. Caregivers' understanding of knowledge related to Alzheimer's disease**

|  | Very familiar | Fairly familiar | Generally familiar | Not very familiar | Completely unfamiliar |
| --- | --- | --- | --- | --- | --- |
| The degree of understanding of AD, N(%) | 33(9.6) | 178(51.6) | 100(29.0) | 25(7.3) | 9(2.6) |
| The degree of understanding of diagnostic tools for AD, N(%) | 20(5.8) | 144(41.7) | 132(38.3) | 40(11.6) | 9(2.6) |
| The degree of understanding of APOE gene testing, N(%) | 23(6.7) | 106(30.7) | 125(36.2) | 69(20) | 22(6.4) |

**Supplementary Table 2. Caregiver Decision-Making Survey**

| ITEM | Proportion(%) |
| --- | --- |
| **Acceptance of diagnostic methods** |  |
| PET | 294(85.2) |
| Cerebrospinal fluid | 94(27.2) |
| Blood | 178(51.6) |
| **The selection of diagnostic methods** |  |
| PET | 315(91.3) |
| Cerebrospinal fluid | 61(17.7) |
| Blood | 69(20) |
| **The factors to pay more attention to before using lecanemab are:** |  |
| Efficacy | 213(66.4) |
| Safety | 102(33.6) |
| Price | 30(10.5) |
| **Reasons for choosing this medication:** |  |
| Doctor's guidance | 286(82.9) |
| Therapeutic effect and demand | 170(49.3) |
| Concerns about the patient's condition progressing to the point where they can no longer take care of themselves | 174(50.4) |
| Related publicity reports | 124(35.9) |
| Concerns about the patient no longer recognizing family members | 136(39.4) |
| **The factors to pay more attention to when using lecanemab are:** |  |
| Safety | 127(40.1) |
| Efficacy | 114(36.0) |
| Price | 78(26.0) |
| Brand | 18(7.5) |
| **Which of the following reasons prompt you to have the patient continue using this medication for treatment?** |  |
| The doctor suggests completing the full course of treatment. | 218(64.9) |
| There are no serious adverse reactions. | 166(49.4) |
| The patient's daily life has improved. | 134(39.9) |
| The patient's symptoms have improved. | 127(37.8) |

**Supplementary Table 3. Caregivers' understanding of lecanemab**

|  | Very familiar | Fairly familiar | Generally familiar | Not very familiar | Completely unfamiliar |
| --- | --- | --- | --- | --- | --- |
| The mechanism of action of lecanemab, N(%) | 30(8.7) | 184(53.3) | 107(31) | 19(5.5) | 5(1.5) |
| The method of lecanemab use and the interval time, N(%) | 106(30.7) | 180(52.2) | 49(14.2) | 8(2.3) | 2(0.6) |
| The price of lecanemab, N(%) | 135(39.1) | 157(45.5) | 40(11.6) | 11(3.2) | 2(0.6) |
| Expected therapeutic effect, N(%) | 35(10.1) | 216(62.6) | 84(24.4) | 7(2.0) | 3(0.9) |
| Adverse reactions, N(%) | 40(11.6) | 218(63.2) | 74(21.5) | 12(3.5) | 1(0.3) |

**Supplementary Table 4. The adverse reactions of lecanemab and their impact on the burden of caregivers**

| Item | Proportion(%) |
| --- | --- |
| Were any treatment-emergent adverse events during lecanemab administration? (Yes) | 67(19.4) |
| Fever | 41(11.9) |
| Dizziness | 19(5.5) |
| Headache | 13(3.8) |
| Changes in caregiving burden following lecanemab initiation |  |
| Worsen | 19(5.5) |
| Physical burden | 16(4.6) |
| Economic burden | 14(4.0) |
| Psychological burden | 11(3.2) |
| Reduce | 89(25.8) |
| Psychological burden | 83(24.0) |
| Physical burden | 51(14.8) |
| Economic burden | 38(11.0) |
| No change | 237(68.7) |

**Supplementary Table 5. Caregivers' Expectations and Suggestions for New DMTs**

| Item | Proportion(%) |
| --- | --- |
| Expected places of using lecanemab |  |
| Hospital wards | 261(75.7) |
| Day infusion centers | 153(44.4) |
| Community clinics | 147(42.6) |
| Actual places |  |
| Hospital wards | 296(85.8) |
| Day infusion centers | 51(14.8) |
| Community clinics | 48(13.9) |
| Expected future dosage forms of DMTs |  |
| Oral | 293(84.9) |
| Subcutaneous injection | 172(49.9) |
| Spray aerosol | 91(26.4) |
| Patch | 98(28.4) |
| Expected intervals of DMTs |  |
| 1 month | 248(71.9) |
| Half a month | 121(35.1) |
| 1 week | 6(1.7) |
| Total duration of using DMTs can accept |  |
| 3 months | 40(11.6) |
| 1 year | 215(62.3) |
| 2 years | 98(28.4) |
| Half a year | 68(19.7) |
| Do you care whether lecanemab is included in the national medical insurance reimbursement scope? Yes | 310(89.8) |

**Supplementary Table 6. Correlation Analysis of Demographic Factors and Caregivers' Confidence in Medication Efficacy**

|  | Extremely | Relatively | Average | Slightly | None | Statistics | P-value |
| --- | --- | --- | --- | --- | --- | --- | --- |
| **Gender** |  |  |  |  |  |  |  |
| Male | 19 (12.6) | 93 (61.6) | 36 (23.8) | 3 (2.0) | 0 (0.0) | 0.503 | 0.615^a^ |
| Female | 19 (9.8) | 137 (70.6) | 35 (18.0) | 2 (1.0) | 1 (0.5) |  |  |
| **Age** |  |  |  |  |  |  |  |
| < 45 | 8 (6.7) | 90 (75.0) | 22 (18.3) | 0 (0.0) | 0 (0.0) | -0.017 | 0.729^b^ |
| 45 - 59 | 16 (12.5) | 78 (60.9) | 29 (22.7) | 4 (3.1) | 1 (0.8) |  |  |
| ≥ 60 | 14 (14.4) | 62 (63.9) | 20 (20.6) | 1 (1.0) | 0 (0.0) |  |  |
| **Education level** |  |  |  |  |  |  |  |
| Tertiary education | 21 (10.9) | 137 (71.0) | 32 (16.6) | 2 (1.0) | 1 (0.5) | 1.357 | 0.175^a^ |
| Non-tertiary education | 17 (11.2) | 93 (61.2) | 39 (25.7) | 3 (2.0) | 0 (0.0) |  |  |
| **Marital status** |  |  |  |  |  |  |  |
| Unmarried | 1 (10.0) | 6 (60.0) | 3 (30.0) | 0 (0.0) | 0 (0.0) | NA | 0.799^c^ |
| Married | 36 (11.2) | 217 (67.2) | 64 (19.8) | 5 (1.6) | 1 (0.3) |  |  |
| Other | 1 (8.3) | 7 (58.3) | 4 (33.3) | 0 (0.0) | 0 (0.0) |  |  |
| **Annual household income(¥10,000)** |  |  |  |  |  |  |  |
| < 20 | 6 (8.3) | 41 (56.9) | 22 (30.6) | 2 (2.8) | 1 (1.4) | -0.157 | 0.001^b^* |
| 20 - 39 | 10 (10.0) | 63 (63.0) | 27 (27.0) | 0 (0.0) | 0 (0.0) |  |  |
| 40 - 59 | 12 (10.5) | 87 (76.3) | 15 (13.2) | 0 (0.0) | 0 (0.0) |  |  |
| > 59 | 10 (17.0) | 39 (66.1) | 7 (11.9) | 3 (5.1) | 0 (0.0) |  |  |
| **Relationship with the patient** |  |  |  |  |  |  |  |
| Adult offspring | 14 (8.2) | 133 (78.2) | 22 (12.9) | 1 (0.6) | 0 (0.0) | NA | 0.001^c^* |
| Spouse | 15 (13.2) | 62 (54.4) | 33 (29.0) | 3 (2.6) | 1 (0.9) |  |  |
| Other | 9 (14.8) | 35 (57.4) | 16 (26.2) | 1 (1.6) | 0 (0.0) |  |  |

a: Cochran-Armitage trend test; b: Kendall rank correlation analysis; c: Fisher's exact test; * indicates P < 0.05; ** indicates P < 0.001

**Supplementary Table 7 Correlation analysis of demographic factors and caregivers' satisfaction with the efficacy of lecanemab**

|  | Very satisfied | Relatively satisfied | Average | Relatively dissatisfied | Very dissatisfied | Statistics | P value |
| --- | --- | --- | --- | --- | --- | --- | --- |
| **Gender** |  |  |  |  |  |  |  |
| Male | 18 (11.9) | 81 (53.6) | 42 (27.8) | 7 (4.6) | 3 (2.0) | 0.617 | 0.538^a^ |
| Female | 11 (5.7) | 111 (57.2) | 65 (33.5) | 5 (2.6) | 2 (1.0) |  |  |
| **Age** |  |  |  |  |  |  |  |
| < 45 | 5 (4.2) | 77 (64.2) | 38 (31.7) | 0 (0.0) | 0 (0.0) | 0.022 | 0.651^b^ |
| 45 - 59 | 11 (8.6) | 68 (53.1) | 40 (31.3) | 6 (4.7) | 3 (2.3) |  |  |
| ≥ 60 | 13 (13.4) | 47 (48.5) | 29 (29.9) | 6 (6.2) | 2 (2.1) |  |  |
| **Education level** |  |  |  |  |  |  |  |
| Tertiary education | 9 (4.7) | 120 (62.2) | 54 (28.0) | 9 (4.7) | 1 (0.5) | 0.080 | 0.936^a^ |
| Non-tertiary education | 20 (13.2) | 72 (47.4) | 53 (34.9) | 3 (2.0) | 4 (2.6) |  |  |
| **Marital status** |  |  |  |  |  |  |  |
| Unmarried | 0 (0.0) | 5 (50.0) | 5 (50.0) | 0 (0.0) | 0 (0.0) | NA | 0.755^c^ |
| Married | 29 (9.0) | 181 (56.0) | 96 (29.7) | 12 (3.7) | 5 (1.6) |  |  |
| Other | 0 (0.0) | 6 (50.0) | 6 (50.0) | 0 (0.0) | 0 (0.0) |  |  |
| **Annual household income(¥10,000)** |  |  |  |  |  |  |  |
| < 20 | 4 (5.6) | 30 (41.7) | 29 (40.3) | 6 (8.3) | 3 (4.2) | -0.235 | <0.001^b^** |
| 20 - 39 | 8 (8.0) | 45 (45.0) | 41 (41.0) | 5 (5.0) | 1 (1.0) |  |  |
| 40 - 59 | 6 (5.3) | 85 (74.6) | 23 (20.2) | 0 (0.0) | 0 (0.0) |  |  |
| > 59 | 11 (18.6) | 32 (54.2) | 14 (23.7) | 1 (1.7) | 1 (1.7) |  |  |
| **Relationship with the patient** |  |  |  |  |  |  |  |
| Adult offspring | 14 (8.2) | 109 (64.1) | 42 (24.7) | 5 (2.9) | 0 (0.0) | NA | 0.040^c^* |
| Spouse | 11 (9.7) | 50 (43.9) | 42 (36.8) | 7 (6.1) | 4 (3.5) |  |  |
| Other | 4 (6.6) | 33 (54.1) | 23 (37.7) | 0 (0.0) | 1 (1.6) |  |  |

a: Cochran-Armitage trend test; b: Kendall rank correlation analysis; c: Fisher's exact test; * indicates P < 0.05; ** indicates P < 0.001

**Supplementary Table 8. Correlation analysis of demographic factors and caregivers' satisfaction with the efficacy of Lecanemab**

|  | Very satisfied | Relatively satisfied | Average | Relatively dissatisfied | Very dissatisfied | Statistics | P value |
| --- | --- | --- | --- | --- | --- | --- | --- |
| **Gender** |  |  |  |  |  |  |  |
| Male | 29 (19.2) | 98 (64.9) | 20 (13.3) | 4 (2.7) | 0 (0.0) | 0.199 | 0.842^a^ |
| Female | 33 (17.0) | 139 (71.7) | 17 (8.8) | 3 (1.6) | 2 (1.0) |  |  |
| **Age** |  |  |  |  |  |  |  |
| < 45 | 12 (10.0) | 92 (76.7) | 16 (13.3) | 0 (0.0) | 0 (0.0) | -0.084 | 0.085^b^ |
| 45 - 59 | 25 (19.5) | 89 (69.5) | 10 (7.8) | 4 (3.1) | 0 (0.0) |  |  |
| ≥ 60 | 25 (25.8) | 56 (57.7) | 11 (11.3) | 3 (3.1) | 2 (2.1) |  |  |
| **Education level** |  |  |  |  |  |  |  |
| Tertiary education | 27 (14.0) | 143 (74.1) | 17 (8.8) | 5 (2.6) | 1 (0.5) | 0.970 | 0.332^a^ |
| Non-tertiary education | 35 (23.0) | 94 (61.8) | 20 (13.2) | 2 (1.3) | 1 (0.7) |  |  |
| **Marital status** |  |  |  |  |  |  |  |
| Unmarried | 2 (20.0) | 3 (30.0) | 5 (50.0) | 0 (0.0) | 0 (0.0) | NA | 0.006^c^* |
| Married | 59 (18.3) | 225 (69.7) | 30 (9.3) | 7 (2.2) | 2 (0.6) |  |  |
| Other | 1 (8.3) | 9 (75.0) | 2 (16.7) | 0 (0.0) | 0 (0.0) |  |  |
| **Annual household income(¥10,000)** |  |  |  |  |  |  |  |
| < 20 | 18 (25.0) | 34 (47.2) | 16 (22.2) | 3 (4.2) | 1 (1.4) | -0.117 | 0.014^b^* |
| 20 - 39 | 11 (11.0) | 72 (72.0) | 13 (13.0) | 3 (3.0) | 1 (1.0) |  |  |
| 40 - 59 | 21 (18.4) | 90 (79.0) | 3 (2.6) | 0 (0.0) | 0 (0.0) |  |  |
| > 59 | 12 (20.3) | 41 (69.5) | 5 (8.5) | 1 (1.7) | 0 (0.0) |  |  |
| **Relationship with the patient** |  |  |  |  |  |  |  |
| Adult offspring | 25 (14.7) | 131 (77.1) | 11 (6.5) | 3 (1.8) | 0 (0.0) | NA | 0.011^c^* |
| Spouse | 26 (22.8) | 67 (58.8) | 15 (13.2) | 4 (3.5) | 2 (1.8) |  |  |
| Other | 11 (18.0) | 39 (63.9) | 11 (18.0) | 0 (0.0) | 0 (0.0) |  |  |

a: Cochran-Armitage trend test; b: Kendall rank correlation analysis; c: Fisher's exact test; * indicates P < 0.05; ** indicates P < 0.001

**Supplementary Table 9. Correlation Analysis of Demographic Factors and caregivers' awareness of Alzheimer's disease**

|  | Very familiar | Fairly familiar | Generally familiar | Not very familiar | Completely unfamiliar | Statistics | P-value |
| --- | --- | --- | --- | --- | --- | --- | --- |
| **Gender** |  |  |  |  |  |  |  |
| Male | 16 (10.6) | 74 (49.0) | 46 (30.5) | 12 (8.0) | 3 (2.0) | 0.003 | 0.997a |
| Female | 17 (8.8) | 104 (53.6) | 54 (27.8) | 13 (6.7) | 6 (3.1) |  |  |
| **Age** |  |  |  |  |  |  |  |
| < 45 | 6 (5.0) | 77 (64.2) | 29 (24.2) | 6 (5.0) | 2 (1.7) | 0.07 | 0.140b |
| 45 - 59 | 14 (10.9) | 61 (47.7) | 43 (33.6) | 8 (6.3) | 2 (1.6) |  |  |
| ≥ 60 | 13 (13.4) | 40 (41.2) | 28 (28.9) | 11 (11.3) | 5 (5.2) |  |  |
| **Education level** |  |  |  |  |  |  |  |
| Tertiary education | 17 (8.8) | 122 (63.2) | 43 (22.3) | 7 (3.6) | 4 (2.1) | 3.61 | <0.001a** |
| Non-tertiary education | 16 (10.5) | 56 (36.8) | 57 (37.5) | 18 (11.8) | 5 (3.3) |  |  |
| **Marital status** |  |  |  |  |  |  |  |
| Unmarried | 2 (20.0) | 3 (30.0) | 5 (50.0) | 0 (0.0) | 0 (0.0) | NA | 0.509c |
| Married | 30 (9.3) | 169 (52.3) | 92 (28.5) | 23 (7.1) | 9 (2.8) |  |  |
| Other | 1 (8.3) | 6 (50.0) | 3 (25.0) | 2 (16.7) | 0 (0.0) |  |  |
| **Annual household income(¥10,000)** |  |  |  |  |  |  |  |
| < 20 | 8 (11.1) | 31 (43.1) | 27 (37.5) | 4 (5.6) | 2 (2.8) | -0.084 | 0.070b |
| 20 - 39 | 8 (8.0) | 44 (44.0) | 34 (34.0) | 12 (12.0) | 2 (2.0) |  |  |
| 40 - 59 | 10 (8.8) | 73 (64.0) | 25 (21.9) | 3 (2.6) | 3 (2.6) |  |  |
| > 59 | 7 (11.9) | 30 (50.9) | 14 (23.7) | 6 (10.2) | 2 (3.4) |  |  |
| **Relationship with the patient** |  |  |  |  |  |  |  |
| Adult offspring | 20 (11.8) | 107 (62.9) | 36 (21.2) | 3 (1.8) | 4 (2.4) | NA | <0.001c** |
| Spouse | 8 (7.0) | 50 (43.9) | 35 (30.7) | 16 (14.0) | 5 (4.4) |  |  |
| Other | 5 (8.2) | 21 (34.4) | 29 (47.5) | 6 (9.8) | 0 (0.0) |  |  |

a: Cochran-Armitage trend test; b: Kendall rank correlation analysis; c: Fisher's exact test; * indicates P < 0.05; ** indicates P < 0.001

**
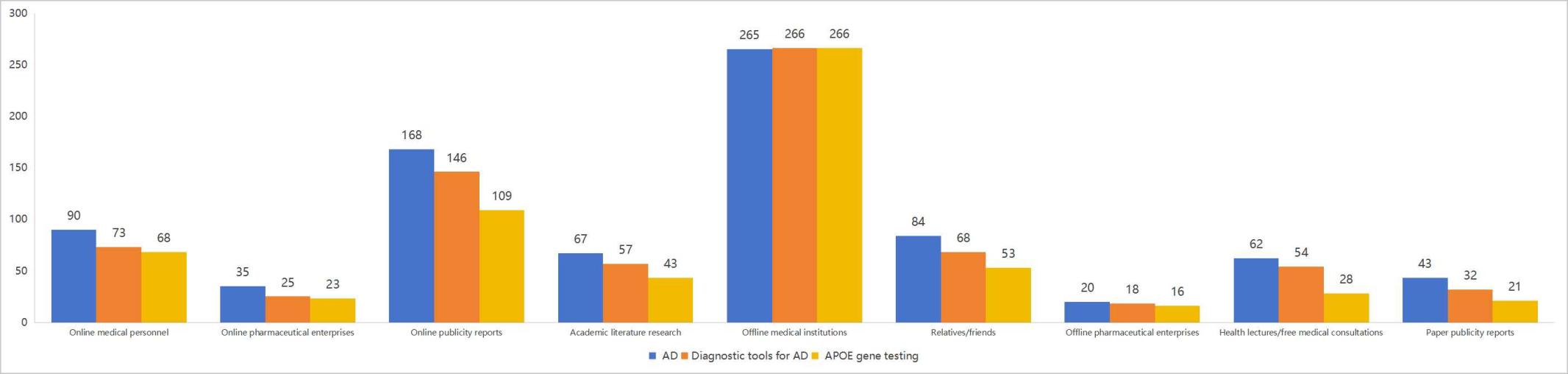
**

**Supplementary Figure1. The access paths for caregivers to acquire knowledge related to Alzheimer's disease (n = 345)**

**
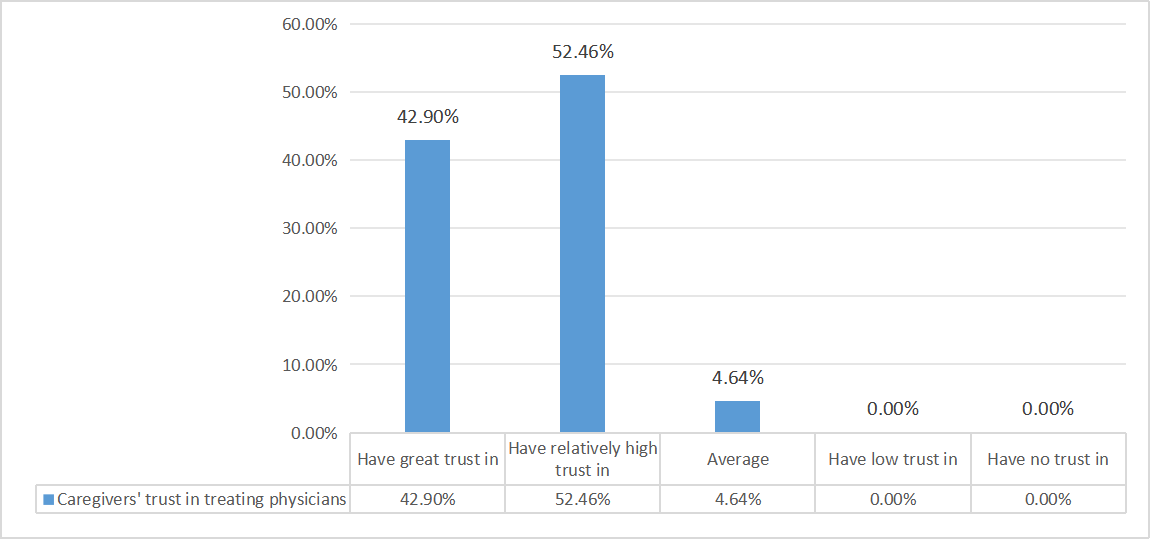
**

**Supplementary Figure2. Caregivers' trust in treating physicians**

**Supplementary Figure3. Caregivers' assessment of patients' adherence to lecanemab treatment**

**Supplementary Figure4. Caregivers' evaluation of the accessibility of lecanemab**
